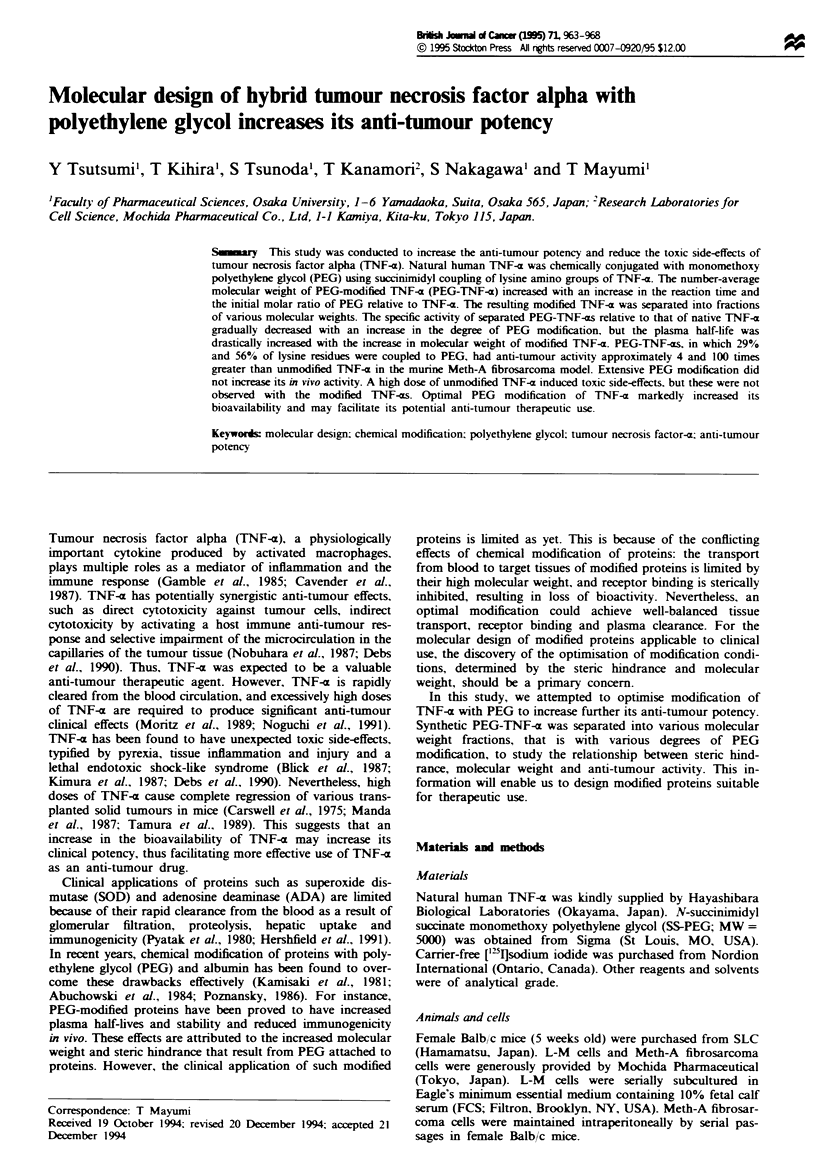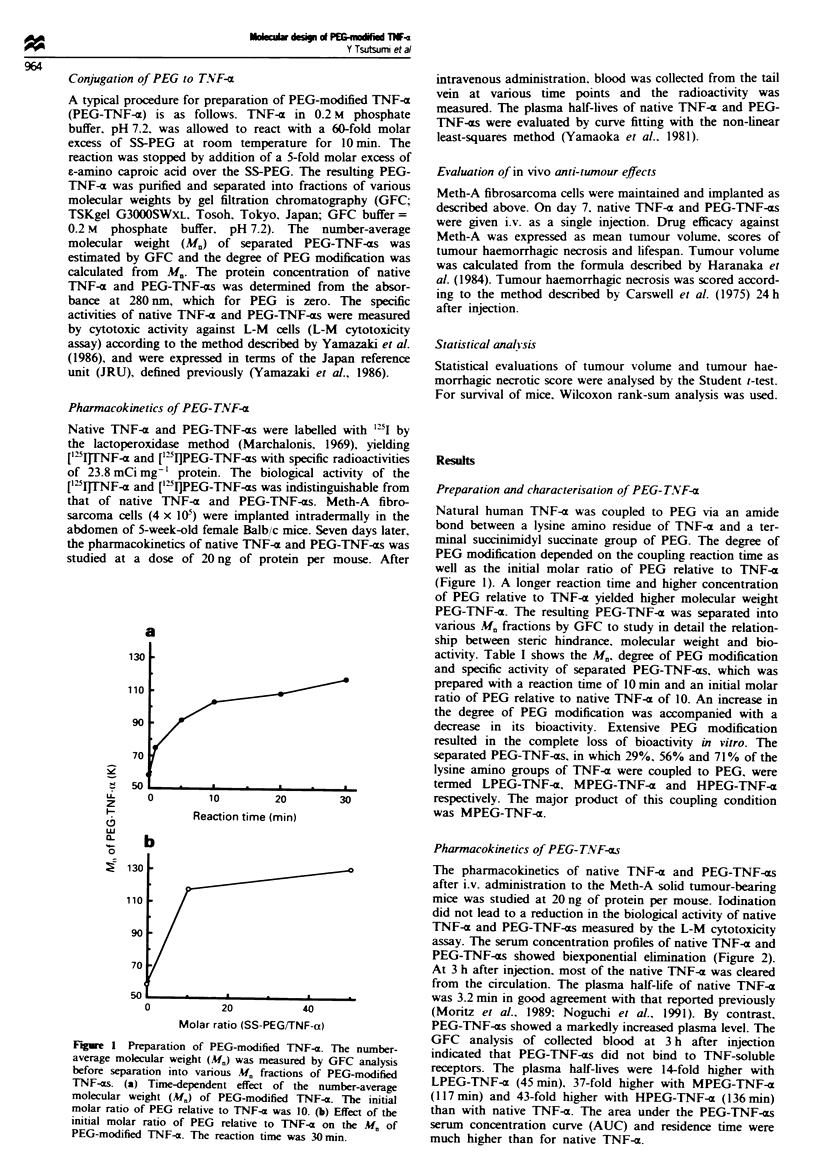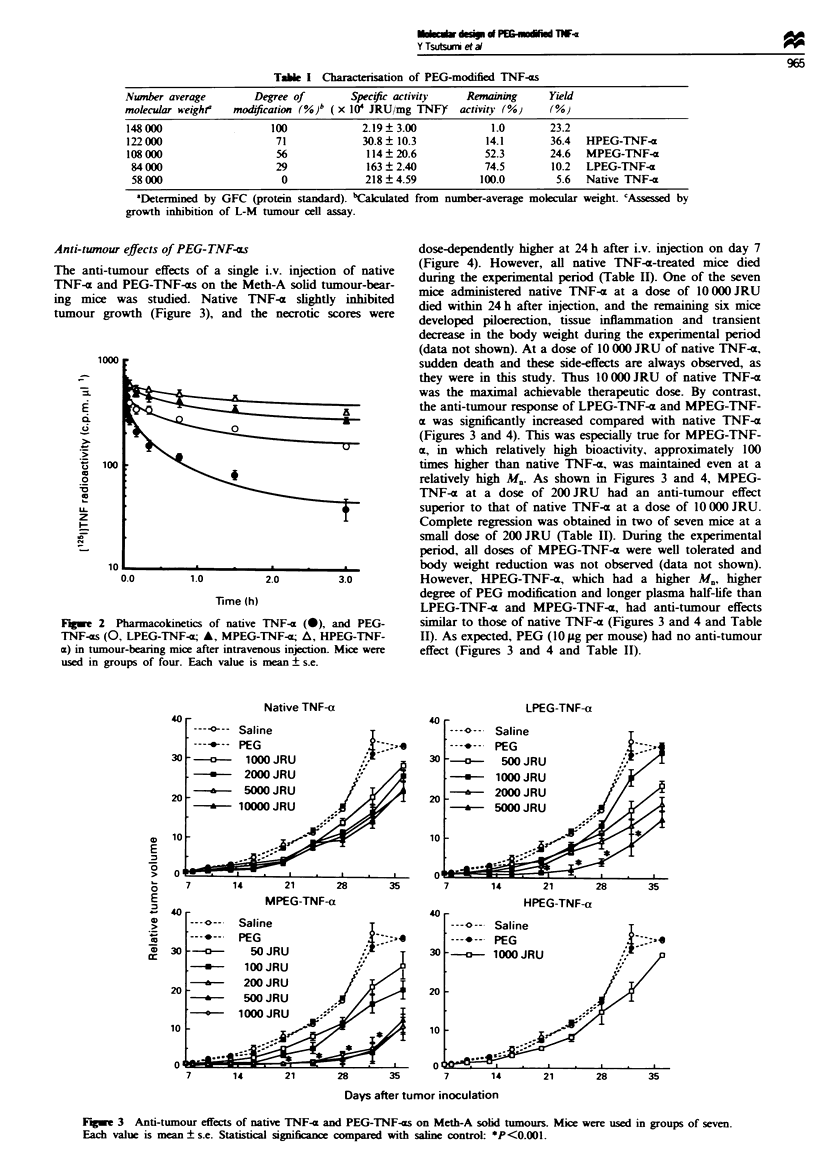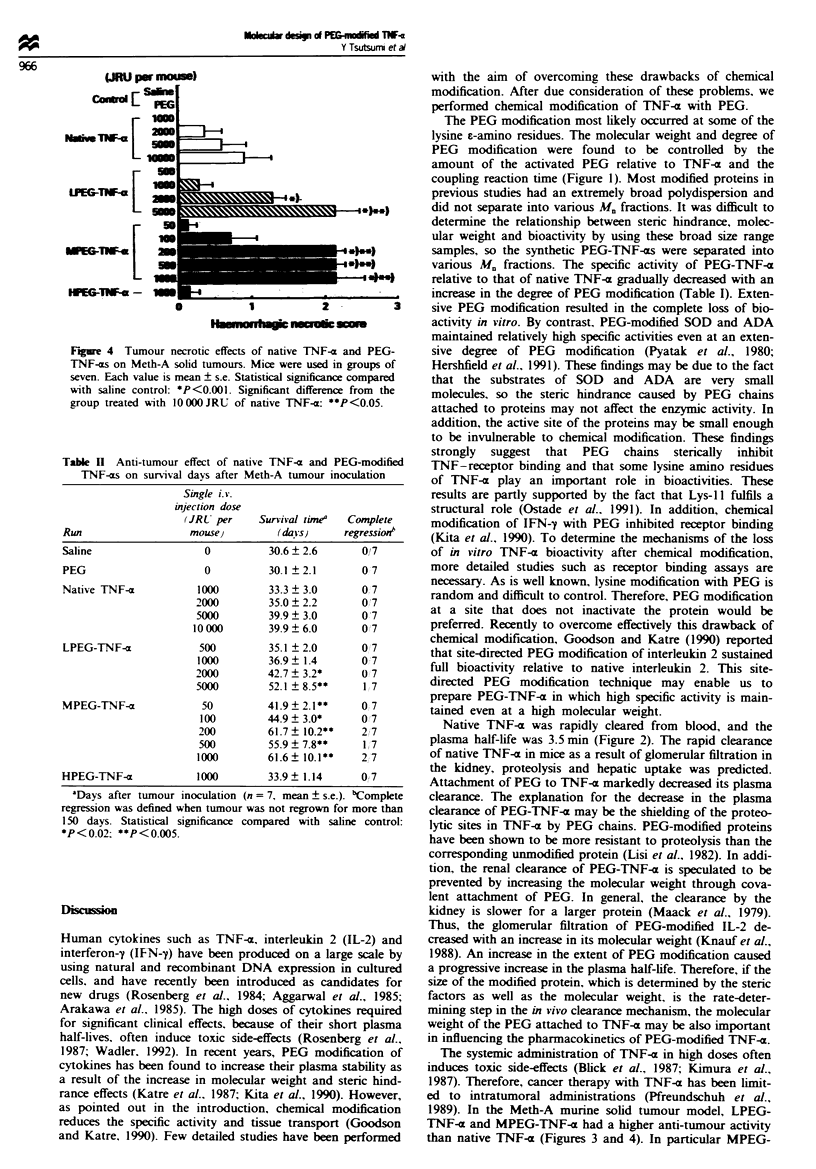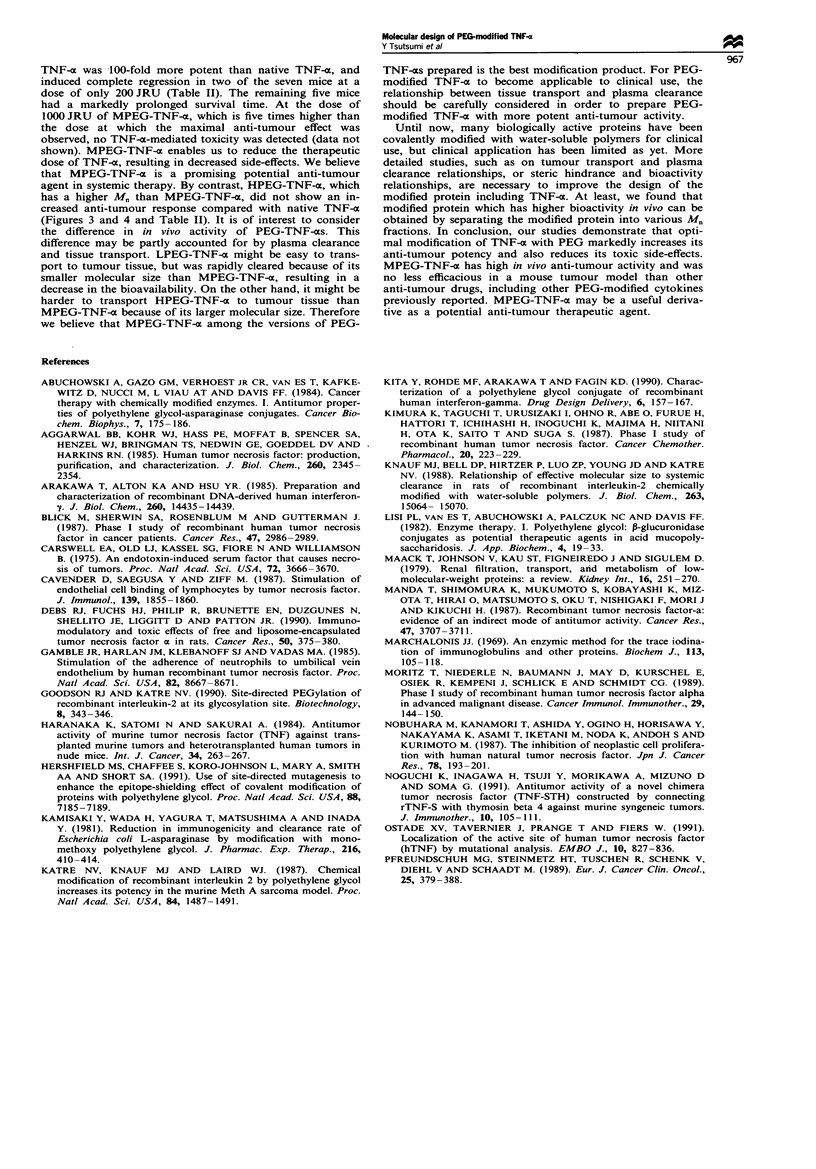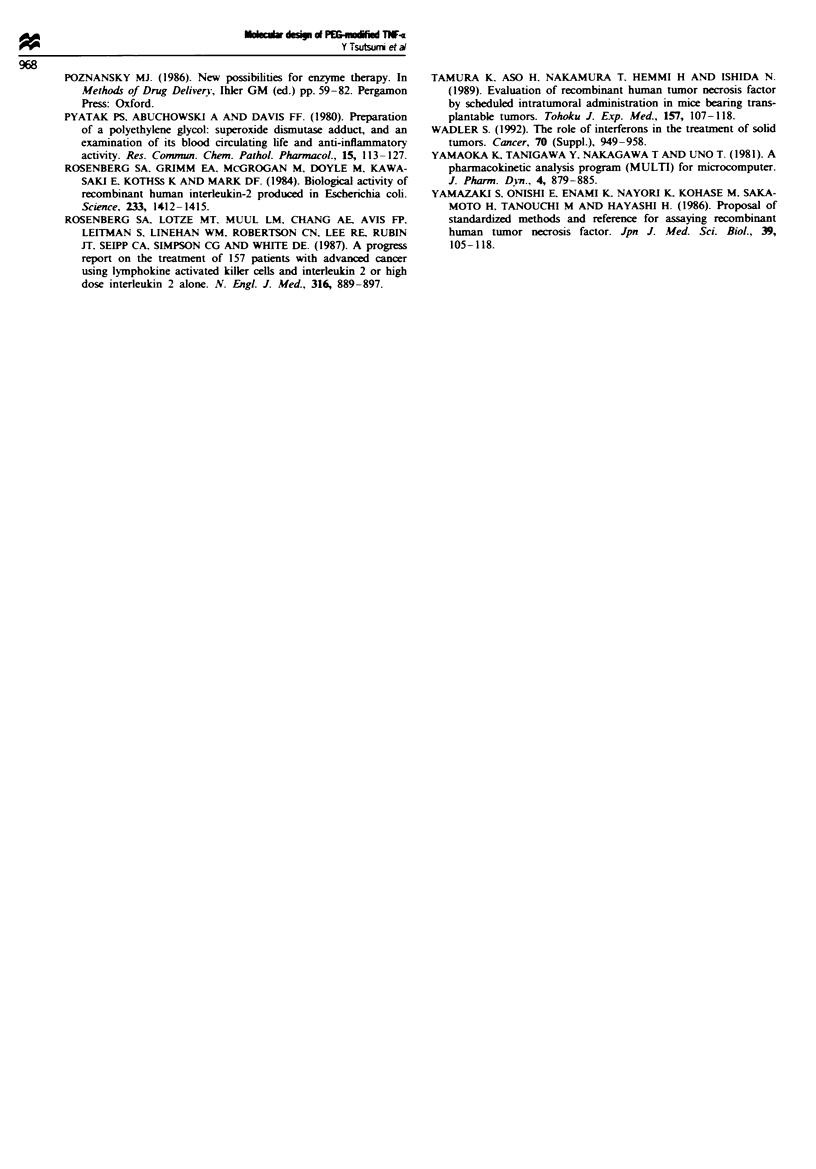# Molecular design of hybrid tumour necrosis factor alpha with polyethylene glycol increases its anti-tumour potency.

**DOI:** 10.1038/bjc.1995.186

**Published:** 1995-05

**Authors:** Y. Tsutsumi, T. Kihira, S. Tsunoda, T. Kanamori, S. Nakagawa, T. Mayumi

**Affiliations:** Faculty of Pharmaceutical Sciences, Osaka University, Japan.

## Abstract

This study was conducted to increase the anti-tumour potency and reduce the toxic side-effects of tumour necrosis factor alpha (TNF-alpha). Natural human TNF-alpha was chemically conjugated with monomethoxy polyethylene glycol (PEG) using succinimidyl coupling of lysine amino groups of TNF-alpha. The number-average molecular weight of PEG-modified TNF-alpha (PEG-TNF-alpha) increased with an increase in the reaction time and the initial molar ratio of PEG relative to TNF-alpha. The resulting modified TNF-alpha was separated into fractions of various molecular weights. The specific activity of separated PEG-TNF-alpha s relative to that of native TNF-alpha gradually decreased with an increase in the degree of PEG modification, but the plasma half-life was drastically increased with the increase in molecular weight of modified TNF-alpha. PEG-TNF-alpha s, in which 29% and 56% of lysine residues were coupled to PEG, had anti-tumour activity approximately 4 and 100 times greater than unmodified TNF-alpha in the murine Meth-A fibrosarcoma model. Extensive PEG modification did not increase its in vivo activity. A high dose of unmodified TNF-alpha induced toxic side-effects, but these were not observed with the modified TNF-alpha s. Optimal PEG modification of TNF-alpha markedly increased its bioavailability and may facilitate its potential anti-tumour therapeutic use.


British Journal of Cancer (1995) 71, 963-968

© 1995 Stockton Press All rights reserved 0007-0920/95 $12.00

### Molecular design of hybrid tumour necrosis factor alpha with polyethylene glycol increases its anti-tumour potency

Y Tsutsumi[1], T Kihira[1], S Tsunoda[1], T Kanamori[2], S Nakagawa[1] and T Mayumi[1]

[1]*Faculty of Pharmaceutical Sciences, Osaka University, 1-6 Yamadaoka, Suita, Osaka 565, Japan;* [2]*Research Laboratories for Cell Science, Mochida Pharmaceutical Co., Ltd, 1-1 Kamiya, Kita-ku, Tokyo 115, Japan.*

**Summary** This study was conducted to increase the anti-tumour potency and reduce the toxic side-effects of tumour necrosis factor alpha (TNF-α). Natural human TNF-α was chemically conjugated with monomethoxy polyethylene glycol (PEG) using succinimidyl coupling of lysine amino groups of TNF-α. The number-average molecular weight of PEG-modified TNF-α (PEG-TNF-α) increased with an increase in the reaction time and the initial molar ratio of PEG relative to TNF-α. The resulting modified TNF-α was separated into fractions of various molecular weights. The specific activity of separated PEG-TNF-αs relative to that of native TNF-α gradually decreased with an increase in the degree of PEG modification, but the plasma half-life was drastically increased with the increase in molecular weight of modified TNF-α. PEG-TNF-αs, in which 29% and 56% of lysine residues were coupled to PEG, had anti-tumour activity approximately 4 and 100 times greater than unmodified TNF-α in the murine Meth-A fibrosarcoma model. Extensive PEG modification did not increase its *in vivo* activity. A high dose of unmodified TNF-α induced toxic side-effects, but these were not observed with the modified TNF-αs. Optimal PEG modification of TNF-α markedly increased its bioavailability and may facilitate its potential anti-tumour therapeutic use.

**Keywords:** molecular design; chemical modification; polyethylene glycol; tumour necrosis factor-α; anti-tumour potency

Tumour necrosis factor alpha (TNF-α), a physiologically important cytokine produced by activated macrophages, plays multiple roles as a mediator of inflammation and the immune response (Gamble *et al.*, 1985; Cavender *et al.*, 1987). TNF-α has potentially synergistic anti-tumour effects, such as direct cytotoxicity against tumour cells, indirect cytotoxicity by activating a host immune anti-tumour response and selective impairment of the microcirculation in the capillaries of the tumour tissue (Nobuhara *et al.*, 1987; Debs *et al.*, 1990). Thus, TNF-α was expected to be a valuable anti-tumour therapeutic agent. However, TNF-α is rapidly cleared from the blood circulation, and excessively high doses of TNF-α are required to produce significant anti-tumour clinical effects (Moritz *et al.*, 1989; Noguchi *et al.*, 1991). TNF-α has been found to have unexpected toxic side-effects, typified by pyrexia, tissue inflammation and injury and a lethal endotoxic shock-like syndrome (Blick *et al.*, 1987; Kimura *et al.*, 1987; Debs *et al.*, 1990). Nevertheless, high doses of TNF-α cause complete regression of various transplanted solid tumours in mice (Carswell *et al.*, 1975; Manda *et al.*, 1987; Tamura *et al.*, 1989). This suggests that an increase in the bioavailability of TNF-α may increase its clinical potency, thus facilitating more effective use of TNF-α as an anti-tumour drug.

Clinical applications of proteins such as superoxide dismutase (SOD) and adenosine deaminase (ADA) are limited because of their rapid clearance from the blood as a result of glomerular filtration, proteolysis, hepatic uptake and immunogenicity (Pyatak *et al.*, 1980; Hershfield *et al.*, 1991). In recent years, chemical modification of proteins with polyethylene glycol (PEG) and albumin has been found to overcome these drawbacks effectively (Kamisaki *et al.*, 1981; Abuchowski *et al.*, 1984; Poznansky, 1986). For instance, PEG-modified proteins have been proved to have increased plasma half-lives and stability and reduced immunogenicity *in vivo*. These effects are attributed to the increased molecular weight and steric hindrance that result from PEG attached to proteins. However, the clinical application of such modified proteins is limited as yet. This is because of the conflicting effects of chemical modification of proteins: the transport from blood to target tissues of modified proteins is limited by their high molecular weight, and receptor binding is sterically inhibited, resulting in loss of bioactivity. Nevertheless, an optimal modification could achieve well-balanced tissue transport, receptor binding and plasma clearance. For the molecular design of modified proteins applicable to clinical use, the discovery of the optimisation of modification conditions, determined by the steric hindrance and molecular weight, should be a primary concern.

In this study, we attempted to optimise modification of TNF-α with PEG to increase further its anti-tumour potency. Synthetic PEG-TNF-α was separated into various molecular weight fractions, that is with various degrees of PEG modification, to study the relationship between steric hindrance, molecular weight and anti-tumour activity. This information will enable us to design modified proteins suitable for therapeutic use.

### Materials and methods

### *Materials*

Natural human TNF-α was kindly supplied by Hayashibara Biological Laboratories (Okayama, Japan). *N*-succinimidyl succinate monomethoxy polyethylene glycol (SS-PEG; MW = 5000) was obtained from Sigma (St Louis, MO, USA). Carrier-free [$^{125}$I]sodium iodide was purchased from Nordion International (Ontario, Canada). Other reagents and solvents were of analytical grade.

### *Animals and cells*

Female Balb/c mice (5 weeks old) were purchased from SLC (Hamamatsu, Japan). L-M cells and Meth-A fibrosarcoma cells were generously provided by Mochida Pharmaceutical (Tokyo, Japan). L-M cells were serially subcultured in Eagle's minimum essential medium containing 10% fetal calf serum (FCS; Filtron, Brooklyn, NY, USA). Meth-A fibrosarcoma cells were maintained intraperitoneally by serial passages in female Balb/c mice.

Correspondence: T Mayumi
Received 19 October 1994; revised 20 December 1994; accepted 21 December 1994

*Conjugation of PEG to TNF-α*

A typical procedure for preparation of PEG-modified TNF-α (PEG-TNF-α) is as follows. TNF-α in 0.2 M phosphate buffer, pH 7.2, was allowed to react with a 60-fold molar excess of SS-PEG at room temperature for 10 min. The reaction was stopped by addition of a 5-fold molar excess of ε-amino caproic acid over the SS-PEG. The resulting PEG-TNF-α was purified and separated into fractions of various molecular weights by gel filtration chromatography (GFC; TSKgel G3000SWXL, Tosoh, Tokyo, Japan; GFC buffer = 0.2 M phosphate buffer, pH 7.2). The number-average molecular weight ($M_n$) of separated PEG-TNF-αs was estimated by GFC and the degree of PEG modification was calculated from $M_n$. The protein concentration of native TNF-α and PEG-TNF-αs was determined from the absorbance at 280 nm, which for PEG is zero. The specific activities of native TNF-α and PEG-TNF-αs were measured by cytotoxic activity against L-M cells (L-M cytotoxicity assay) according to the method described by Yamazaki *et al.* (1986), and were expressed in terms of the Japan reference unit (JRU), defined previously (Yamazaki *et al.*, 1986).

*Pharmacokinetics of PEG-TNF-α*

Native TNF-α and PEG-TNF-αs were labelled with $^{125}$I by the lactoperoxidase method (Marchalonis, 1969), yielding [$^{125}$I]TNF-α and [$^{125}$I]PEG-TNF-αs with specific radioactivities of 23.8 mCi mg$^{-1}$ protein. The biological activity of the [$^{125}$I]TNF-α and [$^{125}$I]PEG-TNF-αs was indistinguishable from that of native TNF-α and PEG-TNF-αs. Meth-A fibrosarcoma cells ($4 \times 10^5$) were implanted intradermally in the abdomen of 5-week-old female Balb/c mice. Seven days later, the pharmacokinetics of native TNF-α and PEG-TNF-αs was studied at a dose of 20 ng of protein per mouse. After intravenous administration, blood was collected from the tail vein at various time points and the radioactivity was measured. The plasma half-lives of native TNF-α and PEG-TNF-αs were evaluated by curve fitting with the non-linear least-squares method (Yamaoka *et al.*, 1981).

**Figure 1** Preparation of PEG-modified TNF-α. The number-average molecular weight ($M_n$) was measured by GFC analysis before separation into various $M_n$ fractions of PEG-modified TNF-αs. (**a**) Time-dependent effect of the number-average molecular weight ($M_n$) of PEG-modified TNF-α. The initial molar ratio of PEG relative to TNF-α was 10. (**b**) Effect of the initial molar ratio of PEG relative to TNF-α on the $M_n$ of PEG-modified TNF-α. The reaction time was 30 min.

*Evaluation of* in vivo *anti-tumour effects*

Meth-A fibrosarcoma cells were maintained and implanted as described above. On day 7, native TNF-α and PEG-TNF-αs were given i.v. as a single injection. Drug efficacy against Meth-A was expressed as mean tumour volume, scores of tumour haemorrhagic necrosis and lifespan. Tumour volume was calculated from the formula described by Haranaka *et al.* (1984). Tumour haemorrhagic necrosis was scored according to the method described by Carswell *et al.* (1975) 24 h after injection.

*Statistical analysis*

Statistical evaluations of tumour volume and tumour haemorrhagic necrotic score were analysed by the Student *t*-test. For survival of mice, Wilcoxon rank-sum analysis was used.

### Results

*Preparation and characterisation of PEG-TNF-α*

Natural human TNF-α was coupled to PEG via an amide bond between a lysine amino residue of TNF-α and a terminal succinimidyl succinate group of PEG. The degree of PEG modification depended on the coupling reaction time as well as the initial molar ratio of PEG relative to TNF-α (Figure 1). A longer reaction time and higher concentration of PEG relative to TNF-α yielded higher molecular weight PEG-TNF-α. The resulting PEG-TNF-α was separated into various $M_n$ fractions by GFC to study in detail the relationship between steric hindrance, molecular weight and bioactivity. Table I shows the $M_n$, degree of PEG modification and specific activity of separated PEG-TNF-αs, which was prepared with a reaction time of 10 min and an initial molar ratio of PEG relative to native TNF-α of 10. An increase in the degree of PEG modification was accompanied with a decrease in its bioactivity. Extensive PEG modification resulted in the complete loss of bioactivity *in vitro*. The separated PEG-TNF-αs, in which 29%, 56% and 71% of the lysine amino groups of TNF-α were coupled to PEG, were termed LPEG-TNF-α, MPEG-TNF-α and HPEG-TNF-α respectively. The major product of this coupling condition was MPEG-TNF-α.

*Pharmacokinetics of PEG-TNF-αs*

The pharmacokinetics of native TNF-α and PEG-TNF-αs after i.v. administration to the Meth-A solid tumour-bearing mice was studied at 20 ng of protein per mouse. Iodination did not lead to a reduction in the biological activity of native TNF-α and PEG-TNF-αs measured by the L-M cytotoxicity assay. The serum concentration profiles of native TNF-α and PEG-TNF-αs showed biexponential elimination (Figure 2). At 3 h after injection, most of the native TNF-α was cleared from the circulation. The plasma half-life of native TNF-α was 3.2 min in good agreement with that reported previously (Moritz *et al.*, 1989; Noguchi *et al.*, 1991). By contrast, PEG-TNF-αs showed a markedly increased plasma level. The GFC analysis of collected blood at 3 h after injection indicated that PEG-TNF-αs did not bind to TNF-soluble receptors. The plasma half-lives were 14-fold higher with LPEG-TNF-α (45 min), 37-fold higher with MPEG-TNF-α (117 min) and 43-fold higher with HPEG-TNF-α (136 min) than with native TNF-α. The area under the PEG-TNF-αs serum concentration curve (AUC) and residence time were much higher than for native TNF-α.

**Table I** Characterisation of PEG-modified TNF-αs

| *Number average molecular weight*[a] | *Degree of modification (%)*[b] | *Specific activity* (× 10[4] JRU/mg TNF)[c] | *Remaining activity (%)* | *Yield (%)* | |
|---|---|---|---|---|---|
| 148 000 | 100 | 2.19 ± 3.00 | 1.0 | 23.2 | |
| 122 000 | 71 | 30.8 ± 10.3 | 14.1 | 36.4 | HPEG-TNF-α |
| 108 000 | 56 | 114 ± 20.6 | 52.3 | 24.6 | MPEG-TNF-α |
| 84 000 | 29 | 163 ± 2.40 | 74.5 | 10.2 | LPEG-TNF-α |
| 58 000 | 0 | 218 ± 4.59 | 100.0 | 5.6 | Native TNF-α |

[a]Determined by GFC (protein standard). [b]Calculated from number-average molecular weight. [c]Assessed by growth inhibition of L-M tumour cell assay.

*Anti-tumour effects of PEG-TNF-αs*

The anti-tumour effects of a single i.v. injection of native TNF-α and PEG-TNF-αs on the Meth-A solid tumour-bearing mice was studied. Native TNF-α slightly inhibited tumour growth (Figure 3), and the necrotic scores were

**Figure 2** Pharmacokinetics of native TNF-α (●), and PEG-TNF-αs (○, LPEG-TNF-α; ▲, MPEG-TNF-α; △, HPEG-TNF-α) in tumour-bearing mice after intravenous injection. Mice were used in groups of four. Each value is mean ± s.e.

dose-dependently higher at 24 h after i.v. injection on day 7 (Figure 4). However, all native TNF-α-treated mice died during the experimental period (Table II). One of the seven mice administered native TNF-α at a dose of 10 000 JRU died within 24 h after injection, and the remaining six mice developed piloerection, tissue inflammation and transient decrease in the body weight during the experimental period (data not shown). At a dose of 10 000 JRU of native TNF-α, sudden death and these side-effects are always observed, as they were in this study. Thus 10 000 JRU of native TNF-α was the maximal achievable therapeutic dose. By contrast, the anti-tumour response of LPEG-TNF-α and MPEG-TNF-α was significantly increased compared with native TNF-α (Figures 3 and 4). This was especially true for MPEG-TNF-α, in which relatively high bioactivity, approximately 100 times higher than native TNF-α, was maintained even at a relatively high $M_n$. As shown in Figures 3 and 4, MPEG-TNF-α at a dose of 200 JRU had an anti-tumour effect superior to that of native TNF-α at a dose of 10 000 JRU. Complete regression was obtained in two of seven mice at a small dose of 200 JRU (Table II). During the experimental period, all doses of MPEG-TNF-α were well tolerated and body weight reduction was not observed (data not shown). However, HPEG-TNF-α, which had a higher $M_n$, higher degree of PEG modification and longer plasma half-life than LPEG-TNF-α and MPEG-TNF-α, had anti-tumour effects similar to those of native TNF-α (Figures 3 and 4 and Table II). As expected, PEG (10 μg per mouse) had no anti-tumour effect (Figures 3 and 4 and Table II).

**Figure 3** Anti-tumour effects of native TNF-α and PEG-TNF-αs on Meth-A solid tumours. Mice were used in groups of seven. Each value is mean ± s.e. Statistical significance compared with saline control: *$P<0.001$.

**Figure 4** Tumour necrotic effects of native TNF-α and PEG-TNF-αs on Meth-A solid tumours. Mice were used in groups of seven. Each value is mean ± s.e. Statistical significance compared with saline control: *$P<0.001$. Significant difference from the group treated with 10 000 JRU of native TNF-α: **$P<0.05$.

**Table II** Anti-tumour effect of native TNF-α and PEG-modified TNF-αs on survival days after Meth-A tumour inoculation

| *Run* | *Single i.v. injection dose (JRU per mouse)* | *Survival time*[a] *(days)* | *Complete regression*[b] |
|---|---|---|---|
| Saline | 0 | 30.6 ± 2.6 | 0/7 |
| PEG | 0 | 30.1 ± 2.1 | 0/7 |
| Native TNF-α | 1000 | 33.3 ± 3.0 | 0/7 |
| | 2000 | 35.0 ± 2.2 | 0/7 |
| | 5000 | 39.9 ± 3.0 | 0/7 |
| | 10 000 | 39.9 ± 6.0 | 0/7 |
| LPEG-TNF-α | 500 | 35.1 ± 2.0 | 0/7 |
| | 1000 | 36.9 ± 1.4 | 0/7 |
| | 2000 | 42.7 ± 3.2* | 0/7 |
| | 5000 | 52.1 ± 8.5** | 1/7 |
| MPEG-TNF-α | 50 | 41.9 ± 2.1** | 0/7 |
| | 100 | 44.9 ± 3.0* | 0/7 |
| | 200 | 61.7 ± 10.2** | 2/7 |
| | 500 | 55.9 ± 7.8** | 1/7 |
| | 1000 | 61.6 ± 10.1** | 2/7 |
| HPEG-TNF-α | 1000 | 33.9 ± 1.14 | 0/7 |

[a]Days after tumour inoculation ($n = 7$, mean ± s.e.). [b]Complete regression was defined when tumour was not regrown for more than 150 days. Statistical significance compared with saline control: *$P<0.02$; **$P<0.005$.

### Discussion

Human cytokines such as TNF-α, interleukin 2 (IL-2) and interferon-γ (IFN-γ) have been produced on a large scale by using natural and recombinant DNA expression in cultured cells, and have recently been introduced as candidates for new drugs (Rosenberg *et al*., 1984; Aggarwal *et al*., 1985; Arakawa *et al*., 1985). The high doses of cytokines required for significant clinical effects, because of their short plasma half-lives, often induce toxic side-effects (Rosenberg *et al*., 1987; Wadler, 1992). In recent years, PEG modification of cytokines has been found to increase their plasma stability as a result of the increase in molecular weight and steric hindrance effects (Katre *et al*., 1987; Kita *et al*., 1990). However, as pointed out in the introduction, chemical modification reduces the specific activity and tissue transport (Goodson and Katre, 1990). Few detailed studies have been performed with the aim of overcoming these drawbacks of chemical modification. After due consideration of these problems, we performed chemical modification of TNF-α with PEG.

The PEG modification most likely occurred at some of the lysine ε-amino residues. The molecular weight and degree of PEG modification were found to be controlled by the amount of the activated PEG relative to TNF-α and the coupling reaction time (Figure 1). Most modified proteins in previous studies had an extremely broad polydispersion and did not separate into various $M_n$ fractions. It was difficult to determine the relationship between steric hindrance, molecular weight and bioactivity by using these broad size range samples, so the synthetic PEG-TNF-αs were separated into various $M_n$ fractions. The specific activity of PEG-TNF-α relative to that of native TNF-α gradually decreased with an increase in the degree of PEG modification (Table I). Extensive PEG modification resulted in the complete loss of bioactivity *in vitro*. By contrast, PEG-modified SOD and ADA maintained relatively high specific activities even at an extensive degree of PEG modification (Pyatak *et al*., 1980; Hershfield *et al*., 1991). These findings may be due to the fact that the substrates of SOD and ADA are very small molecules, so the steric hindrance caused by PEG chains attached to proteins may not affect the enzymic activity. In addition, the active site of the proteins may be small enough to be invulnerable to chemical modification. These findings strongly suggest that PEG chains sterically inhibit TNF–receptor binding and that some lysine amino residues of TNF-α play an important role in bioactivities. These results are partly supported by the fact that Lys-11 fulfils a structural role (Ostade *et al*., 1991). In addition, chemical modification of IFN-γ with PEG inhibited receptor binding (Kita *et al*., 1990). To determine the mechanisms of the loss of *in vitro* TNF-α bioactivity after chemical modification, more detailed studies such as receptor binding assays are necessary. As is well known, lysine modification with PEG is random and difficult to control. Therefore, PEG modification at a site that does not inactivate the protein would be preferred. Recently to overcome effectively this drawback of chemical modification, Goodson and Katre (1990) reported that site-directed PEG modification of interleukin 2 sustained full bioactivity relative to native interleukin 2. This site-directed PEG modification technique may enable us to prepare PEG-TNF-α in which high specific activity is maintained even at a high molecular weight.

Native TNF-α was rapidly cleared from blood, and the plasma half-life was 3.5 min (Figure 2). The rapid clearance of native TNF-α in mice as a result of glomerular filtration in the kidney, proteolysis and hepatic uptake was predicted. Attachment of PEG to TNF-α markedly decreased its plasma clearance. The explanation for the decrease in the plasma clearance of PEG-TNF-α may be the shielding of the proteolytic sites in TNF-α by PEG chains. PEG-modified proteins have been shown to be more resistant to proteolysis than the corresponding unmodified protein (Lisi *et al*., 1982). In addition, the renal clearance of PEG-TNF-α is speculated to be prevented by increasing the molecular weight through covalent attachment of PEG. In general, the clearance by the kidney is slower for a larger protein (Maack *et al*., 1979). Thus, the glomerular filtration of PEG-modified IL-2 decreased with an increase in its molecular weight (Knauf *et al*., 1988). An increase in the extent of PEG modification caused a progressive increase in the plasma half-life. Therefore, if the size of the modified protein, which is determined by the steric factors as well as the molecular weight, is the rate-determining step in the *in vivo* clearance mechanism, the molecular weight of the PEG attached to TNF-α may be also important in influencing the pharmacokinetics of PEG-modified TNF-α.

The systemic administration of TNF-α in high doses often induces toxic side-effects (Blick *et al*., 1987; Kimura *et al*., 1987). Therefore, cancer therapy with TNF-α has been limited to intratumoral administrations (Pfreundschuh *et al*., 1989). In the Meth-A murine solid tumour model, LPEG-TNF-α and MPEG-TNF-α had a higher anti-tumour activity than native TNF-α (Figures 3 and 4). In particular MPEG-

TNF-α was 100-fold more potent than native TNF-α, and induced complete regression in two of the seven mice at a dose of only 200 JRU (Table II). The remaining five mice had a markedly prolonged survival time. At the dose of 1000 JRU of MPEG-TNF-α, which is five times higher than the dose at which the maximal anti-tumour effect was observed, no TNF-α-mediated toxicity was detected (data not shown). MPEG-TNF-α enables us to reduce the therapeutic dose of TNF-α, resulting in decreased side-effects. We believe that MPEG-TNF-α is a promising potential anti-tumour agent in systemic therapy. By contrast, HPEG-TNF-α, which has a higher $M_n$ than MPEG-TNF-α, did not show an increased anti-tumour response compared with native TNF-α (Figures 3 and 4 and Table II). It is of interest to consider the difference in *in vivo* activity of PEG-TNF-αs. This difference may be partly accounted for by plasma clearance and tissue transport. LPEG-TNF-α might be easy to transport to tumour tissue, but was rapidly cleared because of its smaller molecular size than MPEG-TNF-α, resulting in a decrease in the bioavailability. On the other hand, it might be harder to transport HPEG-TNF-α to tumour tissue than MPEG-TNF-α because of its larger molecular size. Therefore we believe that MPEG-TNF-α among the versions of PEG-TNF-αs prepared is the best modification product. For PEG-modified TNF-α to become applicable to clinical use, the relationship between tissue transport and plasma clearance should be carefully considered in order to prepare PEG-modified TNF-α with more potent anti-tumour activity.

Until now, many biologically active proteins have been covalently modified with water-soluble polymers for clinical use, but clinical application has been limited as yet. More detailed studies, such as on tumour transport and plasma clearance relationships, or steric hindrance and bioactivity relationships, are necessary to improve the design of the modified protein including TNF-α. At least, we found that modified protein which has higher bioactivity *in vivo* can be obtained by separating the modified protein into various $M_n$ fractions. In conclusion, our studies demonstrate that optimal modification of TNF-α with PEG markedly increases its anti-tumour potency and also reduces its toxic side-effects. MPEG-TNF-α has high *in vivo* anti-tumour activity and was no less efficacious in a mouse tumour model than other anti-tumour drugs, including other PEG-modified cytokines previously reported. MPEG-TNF-α may be a useful derivative as a potential anti-tumour therapeutic agent.

### References

ABUCHOWSKI A, GAZO GM, VERHOEST JR CR, VAN ES T, KAFKEWITZ D, NUCCI M, L VIAU AT AND DAVIS FF. (1984). Cancer therapy with chemically modified enzymes. I. Antitumor properties of polyethylene glycol-asparaginase conjugates. *Cancer Biochem. Biophys.*, **7**, 175–186.

AGGARWAL BB, KOHR WJ, HASS PE, MOFFAT B, SPENCER SA, HENZEL WJ, BRINGMAN TS, NEDWIN GE, GOEDDEL DV AND HARKINS RN. (1985). Human tumor necrosis factor: production, purification, and characterization. *J. Biol. Chem.*, **260**, 2345–2354.

ARAKAWA T, ALTON KA AND HSU YR. (1985). Preparation and characterization of recombinant DNA-derived human interferon-γ. *J. Biol. Chem.*, **260**, 14435–14439.

BLICK M, SHERWIN SA, ROSENBLUM M AND GUTTERMAN J. (1987). Phase I study of recombinant human tumor necrosis factor in cancer patients. *Cancer Res.*, **47**, 2986–2989.

CARSWELL EA, OLD LJ, KASSEL SG, FIORE N AND WILLIAMSON B. (1975). An endotoxin-induced serum factor that causes necrosis of tumors. *Proc. Natl Acad. Sci. USA*, **72**, 3666–3670.

CAVENDER D, SAEGUSA Y AND ZIFF M. (1987). Stimulation of endothelial cell binding of lymphocytes by tumor necrosis factor. *J. Immunol.*, **139**, 1855–1860.

DEBS RJ, FUCHS HJ, PHILIP R, BRUNETTE EN, DUZGUNES N, SHELLITO JE, LIGGITT D AND PATTON JR. (1990). Immunomodulatory and toxic effects of free and liposome-encapsulated tumor necrosis factor α in rats. *Cancer Res.*, **50**, 375–380.

GAMBLE JR, HARLAN JM, KLEBANOFF SJ AND VADAS MA. (1985). Stimulation of the adherence of neutrophils to umbilical vein endothelium by human recombinant tumor necrosis factor. *Proc. Natl Acad. Sci. USA*, **82**, 8667–8671.

GOODSON RJ AND KATRE NV. (1990). Site-directed PEGylation of recombinant interleukin-2 at its glycosylation site. *Biotechnology*, **8**, 343–346.

HARANAKA K, SATOMI N AND SAKURAI A. (1984). Antitumor activity of murine tumor necrosis factor (TNF) against transplanted murine tumors and heterotransplanted human tumors in nude mice. *Int. J. Cancer*, **34**, 263–267.

HERSHFIELD MS, CHAFFEE S, KORO-JOHNSON L, MARY A, SMITH AA AND SHORT SA. (1991). Use of site-directed mutagenesis to enhance the epitope-shielding effect of covalent modification of proteins with polyethylene glycol. *Proc. Natl Acad. Sci. USA*, **88**, 7185–7189.

KAMISAKI Y, WADA H, YAGURA T, MATSUSHIMA A AND INADA Y. (1981). Reduction in immunogenicity and clearance rate of *Escherichia coli* L-asparaginase by modification with monomethoxy polyethylene glycol. *J. Pharmac. Exp. Therap.*, **216**, 410–414.

KATRE NV, KNAUF MJ AND LAIRD WJ. (1987). Chemical modification of recombinant interleukin 2 by polyethylene glycol increases its potency in the murine Meth A sarcoma model. *Proc. Natl Acad. Sci. USA*, **84**, 1487–1491.

KITA Y, ROHDE MF, ARAKAWA T AND FAGIN KD. (1990). Characterization of a polyethylene glycol conjugate of recombinant human interferon-gamma. *Drug Design Delivery*, **6**, 157–167.

KIMURA K, TAGUCHI T, URUSIZAKI I, OHNO R, ABE O, FURUE H, HATTORI T, ICHIHASHI H, INOGUCHI K, MAJIMA H, NIITANI H, OTA K, SAITO T AND SUGA S. (1987). Phase I study of recombinant human tumor necrosis factor. *Cancer Chemother. Pharmacol.*, **20**, 223–229.

KNAUF MJ, BELL DP, HIRTZER P, LUO ZP, YOUNG JD AND KATRE NV. (1988). Relationship of effective molecular size to systemic clearance in rats of recombinant interleukin-2 chemically modified with water-soluble polymers. *J. Biol. Chem.*, **263**, 15064– 15070.

LISI PL, VAN ES T, ABUCHOWSKI A, PALCZUK NC AND DAVIS FF. (1982). Enzyme therapy. I. Polyethylene glycol: β-glucuronidase conjugates as potential therapeutic agents in acid mucopolysaccharidosis. *J. App. Biochem.*, **4**, 19–33.

MAACK T, JOHNSON V, KAU ST, FIGNEIREDO J AND SIGULEM D. (1979). Renal filtration, transport, and metabolism of low-molecular-weight proteins: a review. *Kidney Int.*, **16**, 251–270.

MANDA T, SHIMOMURA K, MUKUMOTO S, KOBAYASHI K, MIZOTA T, HIRAI O, MATSUMOTO S, OKU T, NISHIGAKI F, MORI J AND KIKUCHI H. (1987). Recombinant tumor necrosis factor-a: evidence of an indirect mode of antitumor activity. *Cancer Res.*, **47**, 3707–3711.

MARCHALONIS JJ. (1969). An enzymic method for the trace iodination of immunoglobulins and other proteins. *Biochem J.*, **113**, 105–118.

MORITZ T, NIEDERLE N, BAUMANN J, MAY D, KURSCHEL E, OSIEK R, KEMPENI J, SCHLICK E AND SCHMIDT CG. (1989). Phase I study of recombinant human tumor necrosis factor alpha in advanced malignant disease. *Cancer Immunol. Immunother.*, **29**, 144–150.

NOBUHARA M, KANAMORI T, ASHIDA Y, OGINO H, HORISAWA Y, NAKAYAMA K, ASAMI T, IKETANI M, NODA K, ANDOH S AND KURIMOTO M. (1987). The inhibition of neoplastic cell proliferation with human natural tumor necrosis factor. *Jpn J. Cancer Res.*, **78**, 193–201.

NOGUCHI K, INAGAWA H, TSUJI Y, MORIKAWA A, MIZUNO D AND SOMA G. (1991). Antitumor activity of a novel chimera tumor necrosis factor (TNF-STH) constructed by connecting rTNF-S with thymosin beta 4 against murine syngeneic tumors. *J. Immunother.*, **10**, 105–111.

OSTADE XV, TAVERNIER J, PRANGE T AND FIERS W. (1991). Localization of the active site of human tumor necrosis factor (hTNF) by mutational analysis. *EMBO J.*, **10**, 827–836.

PFREUNDSCHUH MG, STEINMETZ HT, TUSCHEN R, SCHENK V, DIEHL V AND SCHAADT M. (1989). *Eur. J. Cancer Clin. Oncol.*, **25**, 379–388.

POZNANSKY MJ. (1986). New possibilities for enzyme therapy. In *Methods of Drug Delivery*, Ihler GM (ed.) pp. 59-82. Pergamon Press: Oxford.

PYATAK PS, ABUCHOWSKI A AND DAVIS FF. (1980). Preparation of a polyethylene glycol: superoxide dismutase adduct, and an examination of its blood circulating life and anti-inflammatory activity. *Res. Commun. Chem. Pathol. Pharmacol.*, **15**, 113-127.

ROSENBERG SA, GRIMM EA, MCGROGAN M, DOYLE M, KAWASAKI E, KOTHSS K AND MARK DF. (1984). Biological activity of recombinant human interleukin-2 produced in Escherichia coli. *Science*, **233**, 1412-1415.

ROSENBERG SA, LOTZE MT, MUUL LM, CHANG AE, AVIS FP, LEITMAN S, LINEHAN WM, ROBERTSON CN, LEE RE, RUBIN JT, SEIPP CA, SIMPSON CG AND WHITE DE. (1987). A progress report on the treatment of 157 patients with advanced cancer using lymphokine activated killer cells and interleukin 2 or high dose interleukin 2 alone. *N. Engl. J. Med.*, **316**, 889-897.

TAMURA K, ASO H, NAKAMURA T, HEMMI H AND ISHIDA N. (1989). Evaluation of recombinant human tumor necrosis factor by scheduled intratumoral administration in mice bearing transplantable tumors. *Tohoku J. Exp. Med.*, **157**, 107-118.

WADLER S. (1992). The role of interferons in the treatment of solid tumors. *Cancer*, **70** (Suppl.), 949-958.

YAMAOKA K, TANIGAWA Y, NAKAGAWA T AND UNO T. (1981). A pharmacokinetic analysis program (MULTI) for microcomputer. *J. Pharm. Dyn.*, **4**, 879-885.

YAMAZAKI S, ONISHI E, ENAMI K, NAYORI K, KOHASE M, SAKAMOTO H, TANOUCHI M AND HAYASHI H. (1986). Proposal of standardized methods and reference for assaying recombinant human tumor necrosis factor. *Jpn J. Med. Sci. Biol.*, **39**, 105-118.